# Influence of a Virtual Exercise Program throughout Pregnancy during the COVID-19 Pandemic on Perineal Tears and Episiotomy Rates: A Randomized Clinical Trial

**DOI:** 10.3390/jcm10225250

**Published:** 2021-11-11

**Authors:** Cristina Silva-Jose, Miguel Sánchez-Polán, Ángeles Díaz-Blanco, Tirso Pérez-Medina, Vanessa Carrero Martínez, Irune Alzola, Rubén Barakat, Ignacio Refoyo, Michelle F. Mottola

**Affiliations:** 1 AFIPE Research Group, Faculty of Physical Activity and Sport Sciences-INEF, Universidad Politécnica de Madrid, 28040 Madrid, Spain; cristina.silva.jose@upm.es (C.S.-J.); barakat.ruben@gmail.com (R.B.); 2 Gynecology and Obstetrics Department, Hospital Universitario Severo Ochoa de Leganés, 28911 Leganés, Spain; mariaangelesdiazblanco@gmail.com; 3 Gynecology and Obstetrics Department, Hospital Universitario Puerta de Hierro de Majadahonda, 28222 Majadahonda, Spain; tirso.perez@uam.es (T.P.-M.); vanessacarrerom@gmail.com (V.C.M.); 4 Clínica Zuatzu, 20018 Donostia-San Sebastián, Spain; ialzolae@gmail.com; 5 Sports Department, Faculty of Physical Activity and Sports Sciences-INEF, Universidad Politécnica de Madrid, 28040 Madrid, Spain; ignacio.refoyo@upm.es; 6 R. Samuel McLaughlin Foundation-Exercise and Pregnancy Lab., School of Kinesiology, Faculty of Health Sciences, Department of Anatomy & Cell Biology, Schulich School of Medicine & Dentistry, Children’s Health Research Institute, The University of Western Ontario London, London, ON N6A 3K7, Canada; mmottola@uwo.ca

**Keywords:** pregnancy, pandemic, perineal trauma, exercise

## Abstract

The complications associated with COVID-19 confinement (impossibility of grouping, reduced mobility, distance between people, etc.) influence the lifestyle of pregnant women with important associated complications regarding pregnancy outcomes. Therefore, perineal traumas are the most common obstetric complications during childbirth. The aim of the present study was to examine the influence of a supervised virtual exercise program throughout pregnancy on perineal injury and episiotomy rates during childbirth. A randomized clinical trial design (NCT04563065) was used. Data were collected from 98 pregnant women without obstetric contraindications who attended their prenatal medical consultations. Women were randomly assigned to the intervention (IG, *N* = 48) or the control group (CG, *N* = 50). A virtual and supervised exercise program was conducted from 8–10 to 38–39 weeks of pregnancy. Significant differences were found between the study groups in the percentage of episiotomies, showing a lower episiotomy rate in the IG (*N* = 9/12%) compared to the CG (*N* = 18/38%) (χ^2^ (3) = 4.665; *p* = 0.031) and tears (IG, *N* = 25/52% vs. CG, *N* = 36/73%) (χ^2^ (3) = 4.559; *p* = 0.033). A virtual program of supervised exercise throughout pregnancy during the current COVID-19 pandemic may help reduce rates of episiotomy and perineal tears during delivery in healthy pregnant women.

## 1. Introduction

Perineal traumas are the most common obstetric complications during childbirth [1]. International rates of 85% have been reported for perineal injuries [2], produced either spontaneously, such as tears, or deliberately because of an episiotomy. Recently, the frequency of the appearance of these lesions have been high in Spain, with a prevalence of 59% for tears [3] and between 19% and 50% for episiotomies [4]. The basic function of the episiotomy is to prevent perineal tears due to the tension caused by the mechanisms of labor, especially in primiparous pregnant individuals with instrumental delivery. However, episiotomies also have associated risks to perineal structures.

According to the scientific literature, factors that can affect perineal trauma are primiparity, maternal ethnicity, instrumental delivery (more incidence of forceps than vacuum), birthweight, head circumference, fetal head position or birth duration [5,6,7,8]. Variables that can affect perineal trauma can also negatively affect maternal quality of life [9]. Perineal trauma has been associated with impaired pelvic floor function [10], which is associated with significant short- and long-term morbidity [11,12,13,14]. For example, during the postpartum period, perineal trauma may involve pain and discomfort after birth and dyspareunia, resulting in severe impairment of sexual function [11,15,16]. Urinary and anal incontinence are also reported [14,17] and an increased risk of future pelvic organ prolapse [18]. These physical problems can lead to sleep disorders [19] and increase the risk of postpartum depression [20], thus negatively impacting early motherhood [21].

In addition, relevant public health complications caused by the COVID-19 pandemic (e.g., confinement, mobility restrictions) can become risk factors for numerous pathologies and pregnancy disorders [22,23]. One example is a sedentary lifestyle and decreased movement during COVID restriction that may lead to complications such as an increase in perineal trauma at the time of delivery [24]. The cost to the health care system due to the fact of perineal trauma is important to consider [25].

The relationship between exercise during pregnancy and better delivery results has been previously evidenced, and international guidelines for exercise during pregnancy recommend physical activity for pregnant individuals without obstetric complications [26,27,28]. In fact, previous observational research has shown that maintaining an active lifestyle during pregnancy is associated with a lower incidence of tears during childbirth [3]. Thus, among the mitigating factors of perineal injury, it has been shown that physical activity [7] and physical exercise [29,30] can be used as a preventive agent.

Therefore, during the complex pandemic situation (limitation of face-to-face activities) and the technological boom of virtual modalities, the aim of the current study was to examine the influence of a supervised virtual exercise program throughout pregnancy on perineal injury and episiotomy rates during childbirth. We hypothesized that a supervised, moderate regular exercise program throughout pregnancy using an online model can be a useful factor in controlling and reducing the complications of labor.

## 2. Materials and Methods

### 2.1. Study Design

A randomized clinical trial (registered at “ClinicalTrials.gov”, accessed on 10 June, 2020, registration number: NCT04563065) was conducted via a collaboration between the Obstetrics and Gynecology Department of the Hospital Universitario Severo Ochoa (Madrid), Obstetrics and Gynecology Department of the Hospital Universitario Puerta de Hierro (Madrid), and Clínica Zuatzu and Universidad Politécnica de Madrid. The current study was approved by the Ethical Commission of the Research of Universidad Politécnica de Madrid. 

### 2.2. Participants and Randomization Process

**Figure 1:**
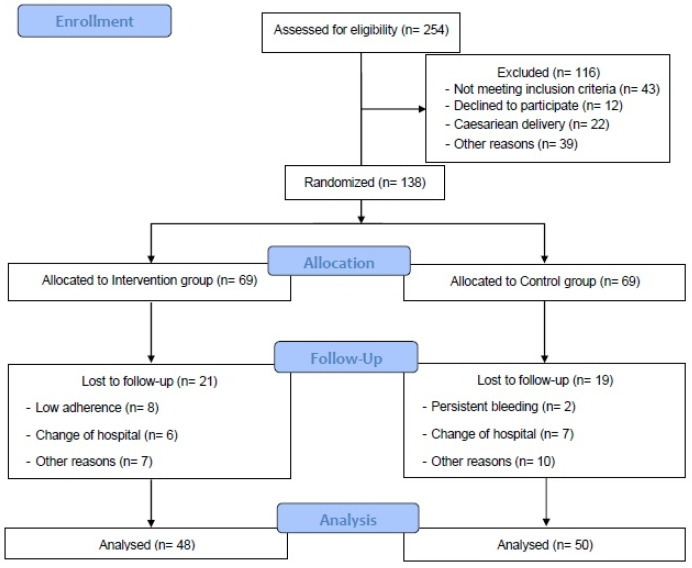
Study population flow chart.

A total of 254 pregnant individuals living in Spain, recruited from hospital obstetric consults (Figure 1), were assessed for eligibility. Individuals between 18 and 42 years with singleton and uncomplicated pregnancies (i.e., type 1, 2, or gestational diabetes at baseline), with no history or risk of preterm delivery were included. Those not planning to give birth in the same obstetric hospital, having a c-section delivery, and not under medical follow up throughout pregnancy were not included, neither were individuals with any serious medical conditions (contraindications) that prevented them from exercising safely [26,27,28].

Participants were randomized to the intervention group (IG) or control group (CG) using REDCap software. A blinded randomization sequence was performed by one researcher using a computer-generated list of random numbers, and it was uploaded to the REDCap database. REDCap performed an arbitrary division of the study participants, using a 1:1 allocation ratio, and all assignments were blinded to hospital staff. Access to the REDCap software for participant randomization was conducted by a different person in each hospital.

### 2.3. Intervention

Pregnant individuals assigned to the intervention group (IG) followed a supervised virtual exercise program throughout pregnancy starting at weeks 8–10. A minimum of 80% adherence was established from a mean total of 80–85 classes planned for each participant for the analysis of results. The exercise program included 3 weekly sessions of 55–60 min of complementary activities following a methodological model divided into seven parts that included pelvic floor exercise established by our research group [31]. Pelvic floor training exercises involved Kegel exercises for 5–10 min each session, which were composed of slow contractions (doing between 2–3 series of 6–8 repetitions and 8 to 10 s each repetition) and fast contractions (doing 1–2 series of 6–8 repetitions and doing 14–18 contractions of 2 to 3 s each repetition) of the different structures of the pelvic floor musculature (vaginal and anal contractions); leg strengthening exercises such as glute bridge or hip abductions; hip mobility exercises, e.g., hip rotations.

The program consisted of: (i) One weekly session of individual work with recorded sessions on a private list in YouTube. These videos were designed with indications and visual information so that pregnant participants could easily follow. (ii) Two group weekly supervised sessions using Zoom software. Classes were offered on separate days to accommodate the participants’ schedules. Participants attended the class for a total of 3 times per week.

Pregnant participants were informed about controlling temperature and humidity to prevent maternal hyperthermia. To control the workload intensity, two mechanisms were used: (i) maternal heart rate (MHR) using a personal monitor; an intensity of 55–65% of the maximum MHR calculated from the Karvonen formula [26,27,28] was reached and maintained during the session. (ii) The perception of effort by Borg’s Rating of Perceived Exertion Scale, using an effort perception of 12–14 (i.e., “somewhat hard”) as a result of the exercise performed [26,27,28]. Prior to starting the program, each participant was instructed on how to determine heart rate range during training and the use of Borg’s scale for perception of exertion. During the workouts, between the different sections, heart rate was recorded by a heart rate monitor or for 10 s at the carotid artery. At the end of the class and taking advantage of the final talk, participants were asked to indicate the perceived effort of the exercises with Borg’s scale.

### 2.4. Control Group

Participants assigned to the CG received normal obstetric health care including materials with physical activity advice or nutritional guidelines throughout pregnancy. To control physical activity, they were asked about exercise once each trimester using a “decision algorithm” (by telephone) [32]. 

### 2.5. Outcomes

All data during and after pregnancy were obtained from hospital records. Maternal and newborn outcomes during pregnancy and childbirth were collected: maternal age, weight, height pre-pregnancy BMI, parity, smoking, occupation, previous miscarriage, type (vaginal c-section) and mode of delivery (instrumentation), perineal tears, episiotomy, birth weight, length, and head circumference. 

### 2.6. Statistical Analysis

Power calculations for the primary outcome (episiotomy) used a prevalence of ~15% in the intervention group and 45–50% in the usual care group [4]. Under these assumptions, a two-sample comparison (χ^2^) with a 5% level of significance and a statistical power of 0.90 gave a study population of 40 patients in each group. Assuming a maximum lost to follow up of 15%, approximately 47 women were needed for each group at baseline [33].

Version 25.0 of IBM SPSS for Windows (IBM Corporation, Armonk, NY, USA) was used. Preliminary assessments were conducted using the Kolmogorov–Smirnov test to screen for violations of normality. Independent *t*-tests were used to assess the differences in age, gestational age, weight, and height between the intervention and control groups. Pearson’s chi-square test was used to compare the frequencies of maternal BMI, smoking, previous miscarriages, parity, and employment occupation between the IG and CG. In addition, this same test was used to determine whether the number and level of perineal tears and episiotomies during childbirth were related to the group and mode of delivery. 

Pairwise correlation coefficients were calculated to examine the interrelationships between maternal outcomes (weight, height, age, and smoking), childbirth outcomes (episiotomy, perineal tear, and mode of delivery), and newborn outcomes (birth weight, birth length, and head circumference). Data for continuous variables are presented as means and standard deviations, and those of the nominal variables are presented as frequencies and percentages. The level of statistical significance was set at *p* < 0.05. 

## 3. Results

A total of 254 women (from 26 September 2020 to 30 June 2021) over 18 years of age were randomized and 116 were excluded: 43 did not meet the inclusion criteria, 12 declined to participate, 22 had caesarean delivery, and 39 for other reasons. Participants were divided into the IG (*n* = 69) and the CG (*n* = 69). In the IG, 21 women were lost to follow up: eight had low adherence, six changed hospitals, and seven for other reasons. In the CG, 19 women were lost to follow up: two had persistent bleeding, seven changed hospitals, and 10 for other reasons. Finally, 48 women in the IG and 50 in the CG were analyzed (Figure 1).

Table 1 shows the general characteristics of the pregnant participants in the study groups. No significant differences (*p* > 0.05) in maternal characteristics were found between the groups at baseline.

The results show that tears appeared in 62.2% and episiotomy in 27.6% of all participants. Pearson’s chi-square test showed significant differences in the number and percentage of tears between the IG (*n* = 48) and CG (*n* = 50) (χ^2^ (3) = 4.559; *p* = 0.033) with the percentage of tears higher in the control group (73% vs. 52%, respectively). 

There were significant differences between the IG and CG (χ^2^ (13) = 12.598; *p* = 0.006) in the type of perineal tears, with 2nd and 3rd degree tears occurring more often in the CG (16% vs. 44% and 0% vs. 4%, respectively) [34]. There was no difference in first-degree perineal tears between groups.

Among pregnant individuals who received an episiotomy, 38% (*N* = 18) were in the CG, whereas 12% (*N* = 9) were in the IG (χ^2^ (3) = 4.665; *p* = 0.031).

In the IG, 44 were non-instrumental and four were instrumental delivery, and in the CG, 42 were non-instrumental and eight were instrumental delivery. In non-instrumental deliveries, there were significant differences in perineal tears (χ^2^ (8) = 7.722; *p* = 0.005) between groups. Of the 42 women in the CG, 34 had perineal tears (82.5%), while there were only 24 of 44 in the IG (54.3%). However, no significant differences were found in the number of episiotomies performed (*p* = 0.256) between the IG (*N* = 7) and the CG (*N* = 10) in the non-instrumental deliveries.

Performing an analysis on instrumental delivery, no significant differences were found (*p* > 0.05) in the appearance of tears between the IG (*N* = 1) and the CG (*N* = 2). On the other hand, significant differences were found (χ^2^ (5) = 4.800; *p* = 0.028), with episiotomies performed in 100% of instrumental deliveries in the control group (*N* = 8), while they were only found in two of four women (50%) in the intervention group.

Table 2 displays the correlation matrix of the IG between maternal, childbirth, and newborn variables. For childbirth variables, episiotomy was negatively associated (*r* = −0.364; *p* = 0.009) and head circumference was positively associated (*r* = 0.370; *p* = 0.026) with the type of perineal tears. Similarly, the mode of delivery was positively associated with birth weight (*r* = 0.342; *p* = 0.020) and length (*r* = 0.378; *p* = 0.023). Head circumference was positively associated with birth weight (*r* = 0.743; *p* < 0.001) and length (*r* = 0.631; *p* < 0.001). Birth weight was positively associated with birth length (*r* = 0.736; *p* < 0.001). Finally, parity was negatively correlated with type of perineal tear (*r* = −0.381; *p* = 0.008) and positively correlated with maternal age (*r* = 0.349; *p* = 0.018).

The correlation matrix in the CG between maternal, childbirth, and newborn variables is shown in Table 3. Episiotomy was negatively correlated with the type of perineal tear (*r* = −0.384; *p* = 0.007). Mode of delivery was positively correlated with episiotomy (*r* = 0.577; *p* < 0.001) and negatively correlated with type of perineal tear (*r* = −0.453; *p* = 0.001). Head circumference was positively associated with birth weight (*r* = 0.641; *p* < 0.001) and length (*r* = 0.471; *p* = 0.009). Moreover, birth weight was positively associated with birth length (*r* = 0.769; *p* < 0.001) and type of perineal tear (*r* = 0.333; *p* = 0.038). 

## 4. Discussion

The main objective of the present study was to examine the influence of a virtual structured exercise program during pregnancy on the appearance and severity of injuries in the perineal area during childbirth. This novel approach used an integration of different types of exercise (resistance, strength, pelvic floor, balance) adopting a group session structure to the online model. These findings suggest that pregnant individuals can successfully participate in online group fitness classes during the ongoing global pandemic. Overall, however, the results showed a disturbing general trend in perineal injuries, with a high percentage (62%) in perineal tears and 28% receiving episiotomies. These types of injuries are especially relevant due to the stress on the pelvic floor [35]. 

The lower occurrence of pelvic floor tears in the IG (73% vs. 52%, respectively) as well as their severity, with 2nd and 3rd degree tears occurring more often in the CG (16% vs. 44% and 0% vs. 4%, respectively) are important findings. In both groups, an episiotomy was negatively correlated with the severity of the perineal tears. This makes sense because surgical incisions may be performed to limit the most serious injuries as has been previously evidenced in the scientific literature [36]. Specific exercise and maintenance of adequate levels of physical activity throughout pregnancy may perhaps protect the perineal area [3].

Regarding non-instrumental delivery, 83% of women in the CG had perineal tears compared to 54% in the IG. On the other hand, of the women with instrumental delivery, all women in the control group (*N* = 8) received an episiotomy, while in the intervention group only two of four women (50%) received an episiotomy. Recently, observational research has shown that the most severe tears and episiotomies occurred more often in the less active group, emphasizing the importance of physical activity pre-pregnancy [37] and during pregnancy [38]. This is unfortunate since the pandemic has forced individuals to limit activity, isolate, and restrict activities [39,40,41]. 

Not surprisingly, the dimensions of newborn birth weight, length, and head circumference were correlated. In the IG, head circumference and, in the CG, birth weight were positively associated with the type of perineal tears, and the greater the head circumference or birth weight, the greater the severity of the tear. Exercise has been shown to normalize fetal growth, preventing macrosomia or excessive growth, which may decrease trauma of the pelvic floor during birth [42,43].

Our results showed additional benefits of an online supervised exercise program in healthy pregnant individuals (including weekly guidelines on a healthy lifestyle), which may be key to reducing childbirth trauma to the pelvic floor and in the immediate postpartum recovery period to prevent comorbidities such as dyspareunia, sexual dysfunction, or urinary incontinence throughout life [44,45,46]. Previous studies have suggested that individuals who practice exercise are less likely to suffer tears with less severity as well as an episiotomy. These investigations were quasi-randomized controlled trials that focused on specific exercises, limiting their intervention to the aquatic environment or Pilates [21,47]. New data on the relationship between maternal exercise and injury during childbirth in the COVID-19 pandemic are needed. In addition, fewer instrumental deliveries and perineal injuries resulted from an active and healthy lifestyle based on face-to-face studies [24,48]. 

We believe this is the first study to link an online supervised exercise intervention with high adherence to the reduction in the number of perineal injuries and severity, with a focus on specific pelvic floor work within exercise sessions promoting an optimal postpartum recovery and maintenance of quality of life. Our RCT confirms the beneficial effects of exercise during pregnancy and demonstrates the importance of using lifestyle-focused treatments as a necessary factor for the prevention of injuries to the pelvic floor and complications during delivery and in the postpartum period. In summary, an online supervised intervention guiding a comprehensive healthy lifestyle may be a necessary and relevant element in the health of the pregnant population during a pandemic.

The strengths of the present study include the inclusion of pelvic floor exercise within a large RCT of an online supervised aerobic exercise intervention with sealed adherence with a minimum of 80% attendance. Furthermore, the physical activity of the CG was measured and controlled (excluding highly active women); however, healthy guideline recommendations were included, since we believe that providing the control group with necessary information is an important part of standard care protocols.

A possible limitation of our study was the lack of nutritional evaluation and education. Nevertheless, all participants received standard care and information on a healthy lifestyle in obstetric care throughout the entire pregnancy. The supervision of a virtual program is not identical to a face-to-face session; however, using online technological resources allowed us to adapt to the pandemic situation. Nonetheless, in the future, we may see more online fitness programs for pregnant individuals and, therefore, future studies should compare the effect of online versus face-to-face supervised group fitness classes on comprehensive maternal and fetal health. Other limitations were that we did not know if the IG completed the other two exercise sessions on their own and if the CG performed a volume of pelvic floor work that could be significant. Finally, the collection of birth information for hospital records may differ depending on the hospital involved.

## 5. Conclusions

A virtual supervised exercise program throughout pregnancy during the ongoing global COVID-19 pandemic reduced injuries to the pelvic floor during childbirth in healthy pregnant women, which may also prevent future comorbidities.

## Figures and Tables

**Table 1:** Maternal characteristics at baseline.

Maternal Characteristics			
Variable	Intervention Group (*n* = 48)	Control Group (*n* = 50)	*p*-Value
**Age (years)**	33.15 ± 4.82	33.54 ± 4.87	0.690
**Maternal height (m)**	1.63 ± 0.05	1.63 ± 0.06	0.630
**Maternal weight (kg)**	61.90 ± 10.57	67.33 ± 18.81	0.085
**BMI (*n*/%)**	22.92 ± 5.10	24.58 ± 3.87	0.072
<18.5	5/10.4	2/4.0	0.330
18.5–24.9	30/62.5	29/58.0	
25–29.9	11/22.9	13/26.0	
>30	2/4.6	6/12.0	
**Parity † (*n*/%)**			
None	31/64.6	36/76.6	0.278
One	11/22.9	9/19.1	
Two or more	6/12.5	2/4.3	
**Smoking during pregnancy**			
No	46/95.8	42/89.4	0.227
Yes	2/4.2	5/10.6	
**Occupation (*n*/%)**			
Active job	26/54.2	20/43.5	0.564
Sedentary job	16/33.3	18/39.1	
Homemaker	6/12.5	8/17.4	
**Previous miscarriage (*n*/%)**			
None	31/64.6	36/73.5	0.076
One	14/29.2	6/12.2	
Two or more	3/6.3	7/14.3	

† Parity: children until current pregnancy.

**Table 2:** Correlation matrix in the IG between maternal, childbirth, and newborn variables.

	Episiotomy	PTT	MD	BW	BL	HC	MA	MW	Parity	Smoking
**Episiotomy**	1									
**PTT**	−0.364 *	1								
**MD**	0.246	−0.072	1							
**BW**	0.168	−0.017	0.342 *	1						
**BL**	0.308	−0.120	0.378 *	0.736 *	1					
**HC**	0.370 *	−0.259	0.197	0.743 *	0.631 *	1				
**MA**	0.030	0.041	−0.026	0.006	−0.042	0.112	1			
**MW**	0.063	−0.182	−0.029	0.259	0.238	0.261	−0.225	1		
**Parity**	0.052	−0.381 *	−0.204	−160	−0.038	−143	0.349 *	0.166	1	
**Smoking**	−0.100	−0.063	−0.063	−0.115	−0.053	−0.279	−0.052	−0.155	0.006	1

BL, birth length; BW, birth weight; HC, head circumference; MA, maternal age; MW, maternal weight; MD, mode of delivery; PTT, perineal tear type. * Statistically significant.

**Table 3:** Correlation matrix in the CG between maternal, childbirth, and newborn variables.

	Episiotomy	PTT	MD	BW	BL	HC	MA	MW	Parity	Smoking
**Episiotomy**	1									
**PTT**	−0.384 *	1								
**MD**	0.577 *	−0.453 *	1							
**BW**	0.145	0.333 *	−0.117	1						
**BL**	0.031	0.127	−0.111	0.769 *	1					
**HC**	0.304	0.049	0.180	0.641 *	0.471 *	1				
**MA**	0.031	−0.099	0.203	0.068	0.021	0.020	1			
**MW**	−0.105	−0.074	0.035	−0.149	−0.116	−0.223	0.112	1		
**Parity**	−0.036	−0.010	0.007	0.201	0.262	0.275	0.209	−0.047	1	
**Smoking**	0.043	0.092	0.049	−0.136	−0.320	−0.327	0.077	−0.118	−0.179	1

BL, birth length; BW, birth weight; HC, head circumference; MA, maternal age; MW, maternal weight; MD, mode of delivery; PTT, perineal tear type. * Statistically significant.

## Data Availability

The data presented in this study are available on request from the corresponding author. The data are not publicly available due to the agreement between the university and participant hospitals.
